# Sequence Relationships of RNA Helicases and Other Proteins Encoded by Blunervirus RNAs Highlight Recombinant Evolutionary Origin of Kitaviral Genomes

**DOI:** 10.3389/fmicb.2020.561092

**Published:** 2020-10-29

**Authors:** Sergey Y. Morozov, Ekaterina A. Lazareva, Andrey G. Solovyev

**Affiliations:** ^1^A. N. Belozersky Institute of Physico-Chemical Biology, Moscow State University, Moscow, Russia; ^2^Department of Virology, Biological Faculty, Moscow State University, Moscow, Russia; ^3^Institute of Molecular Medicine, Sechenov First Moscow State Medical University, Moscow, Russia; ^4^Institute of Agricultural Biotechnology, Moscow, Russia

**Keywords:** kitaviruses, blunerviruses, negeviruses, evolution, RNA helicase, RNA genome, insects, plants

## Indications of Inter-Kingdom RNA Virus Transfer Between Arthropods and Plants

The family *Kitaviridae* includes a small number of positive-stranded RNA plant viruses. Interestingly, this family name is not derived from the name of the type virus member, as usually, but from the family name of Dr. Elliot Watanabe Kitajima, a prominent virologist participating in kitavirus studies (Ramos-González et al., 2020; Quito-Avila et al., in press). Members of the kitavirus genera *Blunervirus, Cilevirus*, and *Higrevirus* produce *in planta* bacilliform (or near spherical) virus particles and have multipartite genomes composed of two, three, or four RNAs for cile-, higre- and bluner-viruses, respectively. In addition to common particle morphology, most viruses share some important biological peculiarities, such as lack of systemic movement (Ramos-González et al., 2020; Quito-Avila et al., in press).

Molecular phylogenetic analyses of the RNA-dependent RNA polymerase (RdRp) domain or concatenated sequences of methyl transferase (MT), replicative RNA helicase (HEL), and RdRp unambiguously showed that kitaviruses are most closely related to members of family *Virgaviridae* [this family name is also not derived from the name of the type virus, like *Bromoviridae*, but from Latin word virga (rod), as all viruses in this family are rod-shaped] followed by those of the family *Bromoviridae* (Quito-Avila et al., 2013; Nunes et al., 2017; Hao et al., 2018; Ramos-González et al., 2020). Accordingly, cileviruses and blunerviruses encode a putative movement protein (MP) with sequence similarity to the MP of *Bromoviridae* (Hao et al., 2018; Quito-Avila et al., in press). Currently, all these viruses are assigned to the recently established positive-stranded RNA virus order *Martellivirales* [this family has been named to honor Prof. Giovanni Paolo Martelli for his contribution to virus taxonomy] (Koonin et al., 2020). However, kitavirus replicative proteins show closest similarity to arthropod-infecting negeviruses (proposed genera Nelorpivirus and Sandewavirus) and nege-like viruses (Kallies et al., 2014; Shi et al., 2016; Nunes et al., 2017; Ramos-González et al., 2020). Recent detailed phylogenetic analysis clearly showed that higrevirus RdRp are closer to cilevirus RdRp than to corresponding blunervirus protein, and these viral RdRp sequences form two separated branches in a phylogenetic dendrogram (Kondo et al., 2020; Ramos-González et al., 2020).

SP24 is the most conserved protein among non-replicative polypeptides encoded by all kitaviruses (Nunes et al., 2017; Ramos-González et al., 2020). Moreover, SP24 was found in negeviruses, many nege-like viruses (Kondo et al., 2020), and in unrelated arthropod-infecting viruses, such as chroparaviruses (Chronic Bee Paralysis Virus) (Kuchibhatla et al., 2014), which, unlike kitaviruses and negeviruses that represent order *Martellivirales*, show genome similarity to arthropod tombus-like viruses (Shi et al., 2016) and belong to separate virus order *Tolivirales* (Koonin et al., 2020). SP24-like proteins (Pfam: 16504) have several trans-membrane domains and a less conserved N-terminal region enriched with positively charged residues. It has been proposed that SP24 molecules could directly interact in virus particles not only with lipids but also with viral RNA due to its positively charged N-terminal region (Kuchibhatla et al., 2014; Solovyev and Morozov, 2017).

Interestingly, two related arthropod viruses with largest genomic RNAs (ca. 16Kb) among nege-like viruses, *Pyrrhocoris apterus* virus 1 (accession MK024711) and Wuhan heteroptera virus 1 (NC_033461) each encode in their monopartite genomes three proteins distantly related to SP24 among seven non-replicative proteins (Supplementary Table 1) (Shi et al., 2016; Koloniuk and Vinokurov, 2019).

The available data on the RdRp and SP24 evolutionary lineages strongly suggest the evolutionary connections between insect nege-like viruses and plant kitaviruses and occurrence of cross-kingdom transfer of positive-stranded RNA viruses between insects and plants during evolution (Kondo et al., 2019, 2020; Ramos-González et al., 2020; Quito-Avila et al., in press). In general, the current views suggest a close temporal parallelism between the evolutionary development of land plants and terrestrial arthropods that started over 450 million years ago and contributed significantly to rapid insect radiation (Kenrick et al., 2012; Labandeira, 2013; Morris et al., 2018). Remarkably, many modern negative-stranded bunyaviruses and rhabdoviruses as well as double-stranded reoviruses using arthropods as vectors have developed an ability to replicate in species from two kingdoms (Whitfield et al., 2018; Chen et al., 2019). These observations agree with the evolutionary inter-kingdom transfer from arthropods to plants recently observed for retrotransposons (Lin et al., 2016; Gao et al., 2018).

It is currently assumed that multiple inter-kingdom jumps between hosts created the modern plant and insect positive-stranded RNA virus diversity, which started its expansion from arthropod hosts as a primary reservoir (Dolja and Koonin, 2018; Shi et al., 2018). In line with this, some present-day insect positive-stranded RNA viruses can replicate in isolated plant cells (like most kitaviruses) or even move systemically in whole plants like tea plant necrotic ring blotch blunervirus (Dasgupta et al., 2001; Annamalai et al., 2008; Hao et al., 2018; Jiwaji et al., 2019). Nege-like plant viruses give us an additional example of inter-kingdom jump and adaptation of typical arthropod-infecting virus to plant host. Indeed, Fragaria vesca-associated virus 1 isolated from plants displaying yellow spot and mosaic symptoms has a single genomic RNA with gene arrangement typical for some nege-like viruses and encodes a replication protein and four non-replicative proteins, two of which are moderately similar to proteins (including SP24) of nege-like viruses, particularly, Hubei Wuhan insect virus 9, barley aphid RNA virus 3, Aphis glycines virus 3 and barley aphid RNA virus 2 (Lenz et al., 2020). Importantly, our previous TBLASTn search of NCBI plant Transcriptome Shotgun Assemblies (TSA) (see Supplementary Figure in Solovyev and Morozov, 2017) revealed several plant-specific “Virus-like RNA assemblies” (VLRAs) encoding replication-like domains moderately similar to nege-like viruses and homologs of SP24 protein (particularly, *Amaranthus tuberculatus* VLRA GGGT01091955; *Humulus lupulus* VLRA GAAW01021049; *Triticum polonicum* VLRA GEDQ01066052).

Outside nege-kitavirus representatives, identification of a unique plant virus in gentian (*Gentiana* sp., asterids; Gentianales; Gentianaceae) strongly argues in favor of quite recent horizontal virus transfer between plant and arthropod hosts. This RNA virus—Gentian Kobu-sho-associated virus (GKaV)—is discovered in a hyperplastic (tumorous disorder-affected) Japanese gentians, possesses a very large genomic RNA (22 Kb) and codes for a polyprotein of more than 810 kDa with prominent sequence similarity to proteins encoded by flaviviruses (Atsumi et al., 2013; Kobayashi et al., 2013). Particularly, phylogeny of viral replicative SF2 (superfamily 2) helicase and RdRp domains revealed most close relation of GKaV to arthropod-infecting flavi-like viruses (Matsumura et al., 2016). Our BLAST analysis of replicative helicase alone also showed that GKaV was most similar to Hermitage virus (accession AMO03217), Apis flavivirus (YP_009388303), Takaungu virus (AMO03219), Lampyris noctiluca flavivirus 1 (QBP37018), Diaphorina citri flavi-like virus (YP_009259672), and Wuhan centipede virus (YP_009254745). It is likely that GKaV is not unique among plant viruses because TBLASTn search of NCBI plant TSA revealed GKaV-related RdRp-coding sequences in *Croton tiglium* (rosids; Malpighiales; Euphorbiaceae) (accession GGDV01007611) (data not shown).

## Novel Putative Blunervirus-Like RNAs in the Transcriptome of *Paulownia tomentosa*

**Figure 1:**
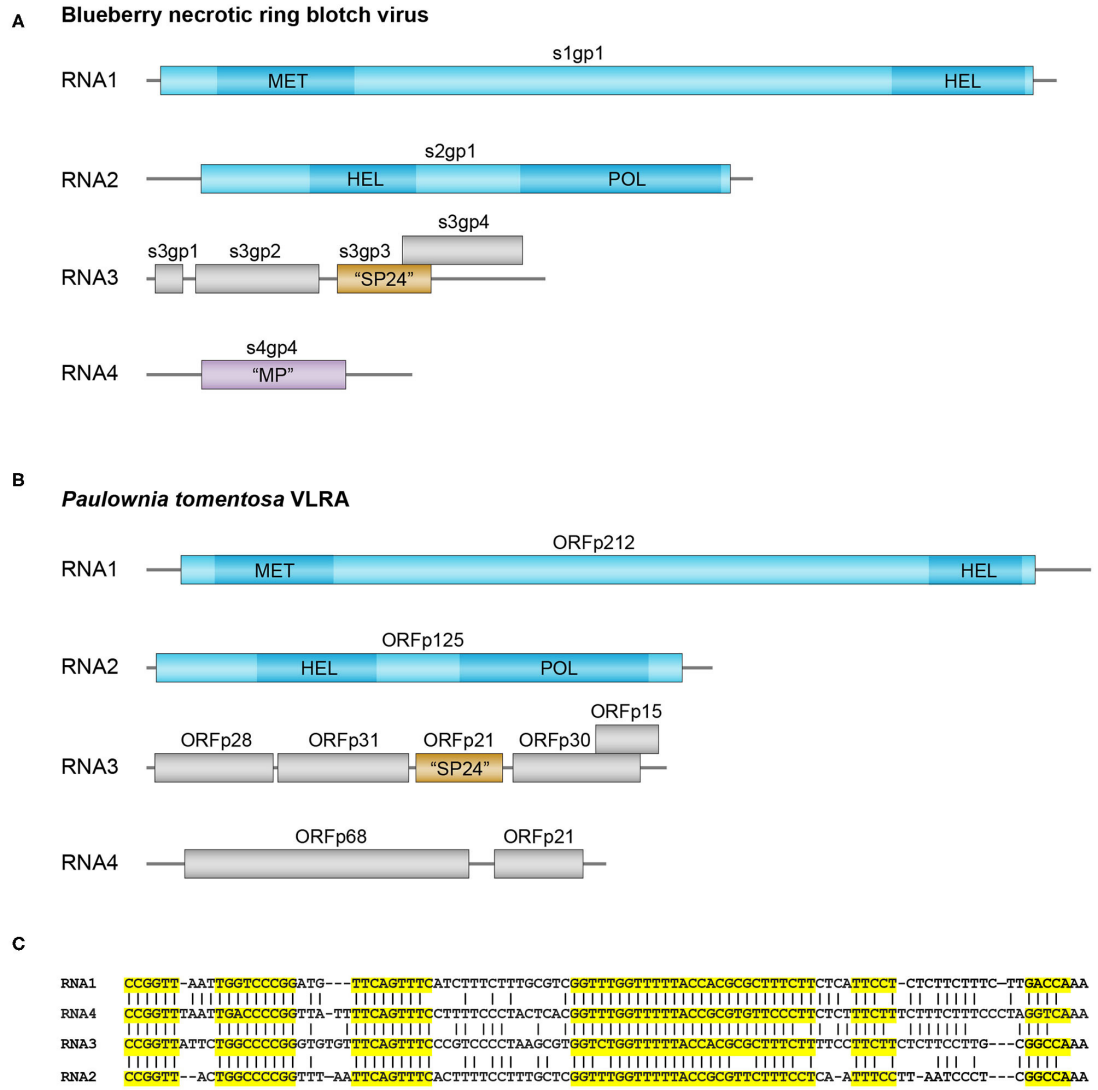
Genomes and sequence analysis of blunerviruses. **(A)** Genome structure of the blueberry necrotic ring blotch blunervirus. Genomic segments (left end corresponds to 5′ end, whereas right end corresponds to 3′ end) encoding putative replication-related proteins are shown in blue, the MET, SF1HEL, and RdRp domains of the putative replication proteins are shown in dark blue. Predicted SP24 ORF protein in segment 3 is in brown. **(B)** Genome structure of the predicted blunervirus from *Paulownia tomentosa*. Putative replication-related genomic segments are shown in blue, the MET, SF1 HEL, and RdRp domains of the putative replication proteins are shown in dark blue. Predicted SP24 ORF protein in segment 3 is in brown. **(C)** Nucleotide sequence alignment of the 3′-terminal regions in the predicted VLRA RNAs from *Paulownia tomentosa*. Highly conserved RNA blocks are highlighted by yellow background.

Our TBLASTn search of very recent NCBI plant TSA data collection resulted in the identification of a new quadripartite set of VLRAs in *Paulownia tomentosa* (dicots, order Lamiales) having a similarity to blunervirus RNAs. Three of these *P. tomentosa* VLRAs exhibit a gene arrangement quite similar to that in RNAs 1, 2, and 3 of blunerviruses (Figures 1A,B). Pairwise BLASTP alignment of the replicative proteins encoded by RNA1 and RNA2 showed their obvious similarity to kitavirus polypeptides (Supplementary Table 1).

In contrast to replicative proteins, most *Paulownia tomentosa* VLRA 3-encoded polypeptides in have only marginal similarity to kitavirus non-replicative proteins. Only SP24 is moderately similar to analogs from kita- and nege-like viruses (Figure 1B and Supplementary Table 1). Other proteins show only rather short amino sequence motifs allowing motif-based sequence comparisons with some kitaviral polypeptides (Supplementary Table 2). Particularly, p31 protein (Figure 1B) contains a rather long C-terminal hydrophobic segment similar to that of cilevirus p61, which exhibits some features of glycoprotein (Leastro et al., 2018), whereas p30 protein includes a common motif with RNA3-encoded p22 of tea plant necrotic ring blotch virus (Supplementary Table 2).

The forth bicistronic *P. tomentosa* VLRA 4 encodes no proteins with significant similarity to other kitavirus non-replicative polypeptides, although pairwise BLASTN analysis of the 3′-untranslated regions from four *P. tomentosa* VLRAs indicated a high degree of sequence conservation among them and strongly suggested that the bicistronic VLRA 4 is indeed the component of the single virus-like multipartite genome (Figure 1C). VLRA 4 proteins could be hardly attributed to viral MPs, because these proteins show no similarity to the blunervirus-encoded orhtologs of well-known MPs found in ilarvirus genomes (*Bromoviridae*) (Quito-Avila et al., 2013, in press; Hao et al., 2018; Bujarski, in press). Instead, VLRA 4 protein p68 (Figure 1B) contains a sequence motif quite similar to the nuclear localization signal (NLS) of cucumoviral 2b proteins known to have silencing suppressor functions (Du et al., 2014; Bujarski, in press) (Supplementary Table 2). Thus it can be speculated that VLRAs discovered by *in silico* analyses of *Paulownia tomoentosa* transcriptome represent a genome of a putative kitavirus that encodes silencing suppressors (Leastro et al., 2020).

Sequence analyses of blunervirus genomes reveal their unusual organization, as two of their four genomic RNAs (RNA1 and RNA2) encode SF1 helicases. Most probably, at least one of the blunervirus SF1 helicases represents a replicative protein. Particularly, RNA1-encoded helicase is a part of protein combining MT and HEL domains similarly to the replicative RNA1-encoded protein in the members of *Bromoviridae* (Hao et al., 2018). According to the phylogenetic trees generated using RdRp sequences, these viruses show relatedness to kitaviruses. Phylogenetic tree showed that all cilevirus and higrevirus RdRp domains are grouped as a single cluster, and RdRp domains of blunervirus replicative proteins also form a separate branch (Kondo et al., 2020; Ramos-González et al., 2020). Accordingly, pairwise sequence comparisons revealed that SF1 helicase domains of Met-HEL and HEL-RdRp proteins encoded by *P. tomentosa* VLRAs like BNRBV Met-HEL helicase are most similar to other blunervirus helicases and nege-like virus helicases (Supplementary Table 1). Strikingly, among six most similar relatives of BNRBV HEL-RdRp helicase SF1 helicases of grapevine leafroll-associated virus 7 (belongs to family *Closteroviridae*), barley aphid RNA negev-like virus 4 and “jiviviruses” (Supplementary Table 1), which is a recently discovered group of positive-stranded plant RNA viruses represented by capsid-less members with three genomic RNAs and named after similar jingmen-like viruses and virga-like viruses (Matsumura et al., 2017; Chiapello et al., 2020). “Jivivirus” RNA1 (around 4 Kb in length) codes for a Met-HEL protein similar in pairwise sequence comparisons to the protein encoded by RNA1 in members of *Bromoviridae* and *Kitaviridae*, whereas RNA2 (around 3 Kb) encodes an RdRp domain also showing relationship to closteroviruses and negev-like viruses (Supplementary Table 1). However, phylogenetic trees of “jivivirus” Met-HEL and RdRp domains revealed obvious similarity to virga-like viruses (Chiapello et al., 2020). Most strikingly, jivivirus RNA3 (around 2 Kb) encodes an SF2 RNA helicase with prominent sequence similarity to flavi-like insect jingmen viruses which contain the respective gene in RNA3 of a quadripartite genome (Chiapello et al., 2020; Garry and Garry, 2020) (Supplementary Figure 1 and Supplementary Table 1). Thus, the structure of “jivivirus” genomes formally corresponds to a reassortant between virga-like and flavi-like viruses and represents a second example among plant viruses when a single genome encodes both SF1 and SF2 RNA helicases. The first such recombinant viral genome has been found for monopartite positive-stranded RNA viruses of the alga genus *Chara*, where SF1 replicative helicase is related to benyvirus proteins, and SF2 helicase is similar to flavi-like viruses (Gibbs et al., 2011; Vlok et al., 2019). Our TBLASTn search of NCBI plant TSA database revealed VLRAs similar to jivivirus genomes in a broad variety of seed plants including members of classes Magnoliopsida, Pinopsida and Ginkgoopsida (Supplementary Table 1 and Legend to Supplementary Figure 1). These results suggest that a recombination/reassortment event between virga-like and flavi-like viruses might have occurred at rather early stages of seed plant evolution.

Based on these results and those presented in our previous papers (Morozov and Solovyev, 2012, 2015; Lazareva et al., 2017), we conclude that it seems logical to consider blunerviruses, and kitaviruses in general, as natural genetic chimeric systems combining in their multipartite genomes viral RNAs of different origin and showing traces of recombination even within individual genomic components.

## Author Contributions

SM collected and analyzed the data and authored drafts of the paper. EL prepared figure and reviewed the final draft. AS authored drafts of the paper, prepared figure, and reviewed the final draft. All authors contributed to the article and approved the submitted version.

## Conflict of Interest

The authors declare that the research was conducted in the absence of any commercial or financial relationships that could be construed as a potential conflict of interest.
