# Supplementary material for: Sequence Relationships of RNA Helicases and Other Proteins Encoded by Blunervirus RNAs Highlight Recombinant Evolutionary Origin of Kitaviral Genomes
Source: Front Microbiol. 2020 Oct 29;11:561092. doi: 10.3389/fmicb.2020.561092 (PMC7658314; doi:10.3389/fmicb.2020.561092)
Supplement: Supplementary file 1 [file Data_Sheet_1.doc]

**Supplementary material**

**Supplementary Fig. 1.** Genome structure of the Grapevine associated jivivirus 1. Virga-like genomic segments are shown in blue, the MET, HEL SF1 and RdRp domains of the putative replication proteins are shown in dark blue, and the proposed HEL SF2 protein in segment 3 is in green. VLRAs encoding proteins quite similar to those of jiviviruses are found in [Magnoliopsida](https://www.ncbi.nlm.nih.gov/Taxonomy/Browser/wwwtax.cgi?mode=Tree&id=3398&lvl=3&lin=f&keep=1&srchmode=1&unlock) **– “**RNA 1” *Aquilaria malaccensis* (GGSI01120237), *Carya illinoinensis* (GGRT01036550), *Mangifera indica* (GHOG01025695 and GHOG01022130), *Persea americana* (GHOF01024838), *Juniperus ashei* (GEWT01001421); “RNA2” *Aquilaria malaccensis* (GGSI01118931), *Carya illinoinensis* (GGRT01040104), *Mangifera indica* (GHOG01027872 and GHOG01019883), *Persea americana* (GHOF01035001), *Juniperus ashei* (GEWT01000483); “RNA3” *Aquilaria malaccensis* (GGSI01121361), *Carya illinoinensis* (QIJ25700), *Mangifera indica* (GHOG01073352 and GHOG01024056), *Persea americana* (GHOF01052815), *Juniperus ashei* (GEWT01001350); Pinopsida - **“**RNA 1” *Pinus albicaulis* (GDQR01018445), *Picea glauca* (GCHX01150549); “RNA2” *Picea glauca* (GCHX01086971); “RNA3” *Picea glauca* (GCHX01360593); [Ginkgoopsida](https://www.ncbi.nlm.nih.gov/Taxonomy/Browser/wwwtax.cgi?mode=Tree&id=29811&lvl=3&lin=f&keep=1&srchmode=1&unlock) **- “**RNA 1” *Ginkgo biloba* (GHLL01290076, GHLL01710728, GHLL01000759, GHLL01469094); “RNA2” *Ginkgo biloba* (GHLL01485824, GHLL01588406, GHLL01588406); “RNA3” *Ginkgo biloba* (GHLL01527468); insects - “RNA2” *Kerria lacca* (GBDO01030160); “RNA3” *Kerria lacca* (GBDO01052225 and GBDO01008782).

**Supplementary Table 1. Pairwise sequence comparisons among the selected proteins encoded by some plus-RNA virus genomes and virus-like transcripts**

| **Query protein** | **Subject ORF** | **E-value** | **Amino acid identity**  **(%)** | **NCBI**  **Accession number** |
| --- | --- | --- | --- | --- |
| **HEL and POL domains** | | | | |
| **Blueberry necrotic ring blotch kitavirus RNA2** | | | | **NC 016085** |
| **SF1 helicase of**  **HEL-RdRp protein** | Citrus virga-like jivivirus RNA1  **Replication protein** | **3e-09** | **29** | **ARO38274** |
|  | Grapevine leafroll-associated velarivirus 7  **Replication protein** | **3e-04** | **28** | **AEQ59443** |
|  | *Aquilaria malaccensis* jivivirus-like VLRA1  **Replication protein** | **1e-27** | **27** | **GGSI01120237** |
|  | *Carya illinoinensis* jivivirus-like VLRA1  **Replication protein** | **8e-08** | **26** | **GGRT01036550** |
|  | *Persea americana* jivivirus-like VLRA1  **Replication protein** | **2e-08** | **26** | **GHOF01024838** |
|  | Barley aphid RNA negev-like virus 4  **Replication protein** | **6e-09** | **25** | **BBV14754** |
| **Blueberry necrotic ring blotch kitavirus RNA1** | | | | **NC 016084** |
| **SF1 helicase of**  **Met-HEL protein** | Tomato blunervirus RNA1  **Met-HEL protein** | **7e-40** | **36** | **QEL52506** |
|  | Tea plant necrotic ring blotch virus RNA1  **Met-HEL protein** | **2e-34** | **35** | **YP_009551524** |
|  | Cordoba negev-like virus  **Replication protein** | **5e-29** | **32** | **AQM55305** |
|  | Wuhan insect negev-like virus 8  **Replication protein** | **6e-28** | **33** | **YP_009344994** |
|  | Culex negev-like virus 2  **Replication protein** | **5e-27** | **33** | **YP_009388582** |
| ***Paulownia tomentosa* Blunervirus-like VLRA1** | | | | **GEFV01158142** |
| **SF1 helicase of**  **Met-HEL protein** | Tomato blunervirus RNA1  **Met-HEL protein** | **1e-30** | **37.3** | **QEL52506** |
|  | Blueberry necrotic ring blotch virus RNA1  **Met-HEL protein** | **4e-33** | **35.8** | **AGI44297** |
|  | Wuhan insect negev-like virus 8  **Replication protein** | **1e-29** | **37** | **YP_009344994** |
|  | Brejeira negevirus  **Replication protein** | **3e-28** | **37** | **AQM55484** |
| ***Paulownia tomentosa* Blunervirus-like VLRA2** | | | | **GEFV01018191** |
| **SF1 helicase of**  **HEL-RdRp protein** | Tomato blunervirus RNA2  **HEL-RdRp protein** | **2e-29** | **39** | **QEL52507** |
|  | Beihai barnacle negev-like virus 2  **Replication protein** | **3e-30** | **37** | **YP_009333216** |
|  | Wuhan insect negev-like virus 8  **Replication protein** | **7e-28** | **34** | **YP_009344994** |
|  | Blueberry necrotic ring blotch virus RNA1  **Met-HEL protein** | **3e-27** | **36** | **AGI44297** |
| **Grapevine associated jivivirus 1 RNA1** | | | | **MN520745** |
| **Met-HEL**  **Replication SF1 helicase** | Citrus virga-like jivivirus RNA1  **Replication SF1 HEL** | **4e-48** | **46** | **ARO38274** |
|  | Grapevine associated jivivirus 2 RNA1  **Replication SF1 HEL** | **1e-43** | **42** | **QIJ25701** |
|  | Aquilaria malaccensis VLRA RNA1  **Replication SF1 HEL** | **2e-65** | **49** | **GGSI01120237** |
|  | Carya illinoinensis VLRA RNA1 **Replication SF1 HEL** | **1e-60** | **47** | **GGRT01036550** |
|  | Picea glauca VLRA RNA1  **Replication SF1 HEL** | **1e-47** | **42.2** | **GCHX01150549** |
|  | Pea early-browning tobravirus RNA  **Replication SF1 HEL** | **3e-20** | **39** | [**BCA87380**](https://www.ncbi.nlm.nih.gov/protein/BCA87380.1?report=genbank&log$=protalign&blast_rank=5&RID=AAH8SM3G014) |
|  | Beet soil-borne pomovirus RNA1  **Replication SF1 HEL** | **2e-18** | **35** | **QCF59318** |
| **Grapevine associated jivivirus 1 RNA2** | | | | **QIJ25699** |
| **RdRp domain** | Citrus virga-like jivivirus RNA3  **RdRp domain** | **5e-103** | **55** | **ARO38275** |
|  | Grapevine associated jivivirus 2 RNA2  **RdRp domain** | **2e-75** | **45** | **QIJ25702** |
|  | Carya illinoinensis VLRA RNA2  **RdRp domain** | **1e-126** | **63** | **GGRT01040104** |
|  | Aquilaria malaccensis VLRA RNA2  **RdRp domain** | **2e-114** | **60.5** | **GGSI01118931** |
|  | Picea glauca VLRA RNA2  **RdRp domain** | **3e-79** | **45** | **GCHX01086971** |
|  | Plum bark necrosis stem pitting-associated ampelovirus  **RdRp domain** | **2e-28** | **32** | **BCA25965** |
|  | Persimmon ampelovirus  **RdRp domain** | **8e-27** | **30** | **BBL52483** |
|  | Negev virus  **RdRp domain** | **4e-24** | **32** | **BAR91505** |
|  | Atrato Virga-like virus 2  **RdRp domain** | **2e-24** | **32** | **QHA33740** |
| **Grapevine associated jivivirus 1 RNA3** | | | | **MN520747** |
| **SF2 helicase** | Citrus jingmen-like virus  **SF2 HEL** | **1e-81** | **58** | **ARO38273** |
|  | Grapevine associated jivivirus 2  **SF2 HEL** | **1e-42** | **39** | **QIJ25703** |
|  | Carya illinoinensis VLRA RNA3  **SF2 HEL** | **4e-82** | **57** | **QIJ25700** |
|  | Aquilaria malaccensis VLRA RNA3  **SF2 HEL** | **2e-86** | **61** | **GGSI01121361** |
|  | Picea glauca VLRA RNA3  **SF2 HEL** | **1e-44** | **41** | **GCHX01360593** |
|  | Kerria lacca** VLRA3  **SF2 HEL** | **5e-116** | **54** | **GBDO01052225**  **GBDO01008782** |
|  | Jingmen tick virus***  **NS3-like protein** | **4e-08** | **31** | **AHZ31672** |
| **SP24-like proteins*** | | | | |
| **Brandeis negev-like virus** | | | | **AVZ66285** |
| **SP24 domain** | Hubei virga-like virus 18  **gp3 protein** | **1e-62** | **47** | **YP_009333288** |
|  | Culex negev-like virus 1  **gp3 protein** | **8e-17** | **31** | **AXV43885** |
|  | Aedes albopictus negev-like virus  **ORF3 protein** | **2e-16** | **31** | **QGN03451** |
|  | Pyrrhocoris apterus virus 1  **22K protein gp4** | **1e-08** | **26** | **AYH53284** |
|  | Wuhan heteroptera virus 1  **23K protein gp5** | **2e-09** | **28** | **EU330453** |
|  | Pyrrhocoris apterus virus 1  **26K protein gp5** | **1e-08** | **29** | **AYH53285** |
|  | Wuhan heteroptera virus 1  **26K protein gp6** | **1e-03** | **26** | **YP_009342334** |
|  | Pyrrhocoris apterus virus 1  **56K protein gp6** | **1e-08** | **29** | **AYH53286** |
|  | Wuhan heteroptera virus 1  **58K protein gp7** | **1e-18** | **26** | **YP_009342335** |
| ***Paulownia tomentosa* VLRA3 gp3** | | | | **GEFV01018861** |
| **SP24 domain** | Blueberry necrotic ring blotch virus  **SP24** | **2e-22** | **39** | **YP_004901704** |
|  | Tomato blunervirus 1  **SP24** | **4e-15** | **31** | **QEL52510** |
|  | Tea plant necrotic ring blotch virus  **SP24** | **7e-12** | **28** | **YP_009551528** |
|  | Negev virus  **gp3** | **2e-06** | **29** | **YP 009256207** |

* - two query proteins under this section of table

** - this is VLRA from insect source (order Hemiptera)

*** - this virus infects hosts of class Arachnida (order Ixodida) (Qin et al., 2014)

**Supplementary Table 2. Motif comparisons among the selected proteins encoded by some positive-stranded RNA virus genomes and virus-like transcripts**

| **Protein and motif position** | **Motif sequence** | **Proportion of hydrophobic residues** | **Charged residues** | **Reference and**  **accession number** |
| --- | --- | --- | --- | --- |
| ***Paulownia tomentosa* VLRA3 p31 (197-281)** | **EHLVQSLITTVVRTLFSVLQGAISHLVHFAFPPISELFHVLEQLFDACVPFGVLEGVFFALVCCRFAHPYVVTVFAFVVALVSVK** | **53/85** | **8/85** | **This paper (GEFV01018861)** |
| **Citrus leprosis virus C p61 (451-521)** | **eclstvfkvlfsaigaclsfivdvggccfrhfifvcldsvillllllptythltfilgftlnayiqlvyfe** | **47/71** | **6/71** | **Locali-Fabris et al., 2006* (NC_008170)** |
| ***Paulownia tomentosa* VLRA3 p30 (216-236)** | **NRRRRDVGDALAEVADTVGDV** | **8/21** | **9/21** | **This paper (GEFV01018861)** |
| **Blueberry necrotic ring blotch virus p28 (194-214)** | **SRGKRDVSRIVAGVADSVGDV** | **8/21** | **7/21** | **Cantu-Iris et al., 2013****  **(AGI44302)** |
| **Tea plant necrotic ring blotch virus p22 (143-163)** | **TRRRRSVEGTVSEIADTVGGV** | **6/21** | **7/21** | **Hao et al., 2018**  **(YP_009551529)** |
| ***Paulownia tomentosa* VLRA4 p68 (144-157)** | **AAPSHRRRHRRGRRNKG** | **3/17** | **8/17** | **This paper (GEFV01018726)** |
| **Cucumber mosaic virus 2b NLS-bearing protein (21-37)** | **VKRhrrrshkknrrerg** | **1/17** | **10/17** | **Du et al., 2014**  **(AAR89476)** |
| **Peanut stunt virus 2b protein (17-33)** | **KQKSARRRHRQNRKARG** | **2/17** | **9/17** | **Netsu et al., 2008*****  **(BAF80087)** |

Yellow shading indicates GxxxG-like sequence motifs (GxxxG, GxxxA, SxxxG, etc.) that can mediate protein hetero- and oligomerization involving intramembrane protein-protein contacts [Fink, A., Sal-Man, N., Gerber, D., Shai, Y. (2012). [Transmembrane domains interactions within the membrane milieu: principles, advances and challenges.](https://pubmed.ncbi.nlm.nih.gov/22155642/) *Biochim. Biophys. Acta* 1818, 974-983. doi: 10.1016/j.bbamem.2011.11.029].

* - Locali-Fabris, E. C., Freitas-Astua, J., Souza, A. A., Takita, M. A., Astua-Monge, G., Antonioli-Luizon, R., Rodrigues, V., Targon,M. L., and Machado, M.A. (2006). Complete nucleotide sequence, genomic organization and phylogenetic analysis of Citrus leprosis virus cytoplasmic type. *J. Gen. Virol*. 87, 2721-2729.

** - Cantu-Iris, M., Harmon, P.F., Londono, A. and Polston, J.E. (2013). A variant of blueberry necrotic ring blotch virus associated with red lesions in blueberry. *Arch. Virol*. 158, 2197-2200.

*** - Netsu, O., Hiratsuka, K., Kuwata, S., Hibi, T., Ugaki, M. and Suzuki, M. (2008). Peanut stunt virus 2b cistron plays a role in viral local and systemic accumulation and virulence in Nicotiana benthamiana. *Arch. Virol*. 153, 1731-1735.
