# Supplementary figures and images for: Sequence Relationships of RNA Helicases and Other Proteins Encoded by Blunervirus RNAs Highlight Recombinant Evolutionary Origin of Kitaviral Genomes
Source: Front Microbiol. 2020 Oct 29;11:561092. doi: 10.3389/fmicb.2020.561092 (PMC7658314; doi:10.3389/fmicb.2020.561092)

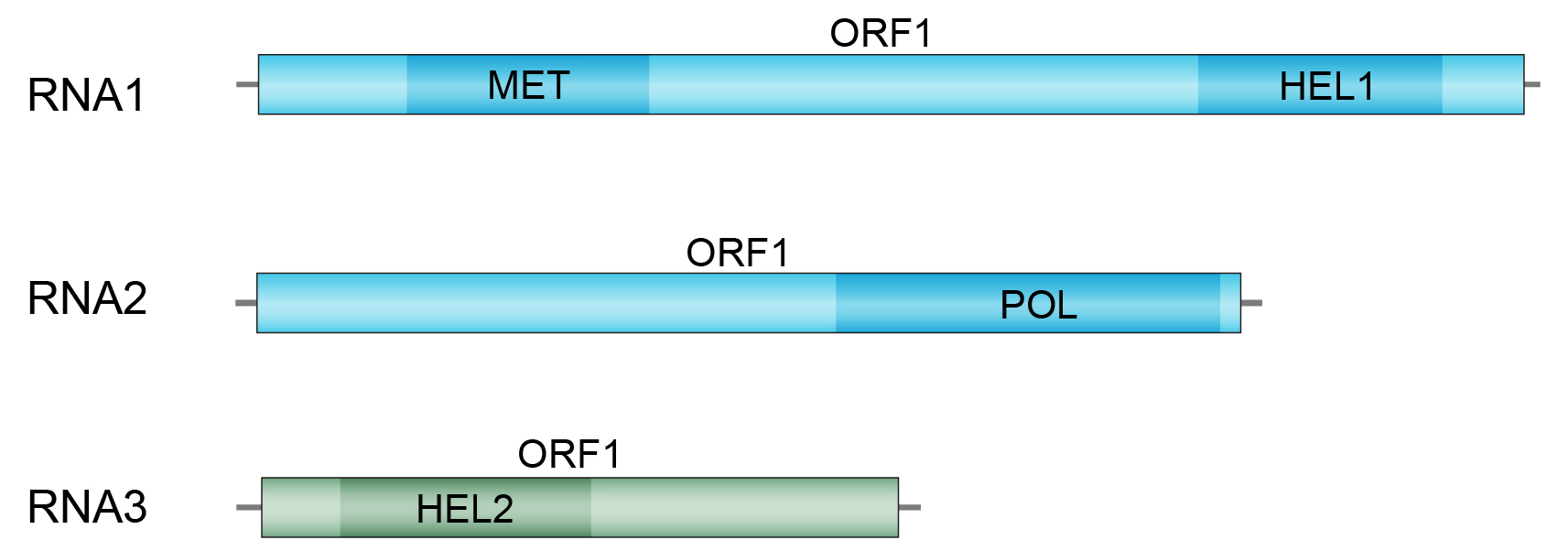

Supplement: Supplementary file 2 [file Image_1.jpg]
